# Supplementary material for: Association between oral microbiome and five types of respiratory infections: a two-sample Mendelian randomization study in east Asian population
Source: Front Microbiol. 2024 Apr 10;15:1392473. doi: 10.3389/fmicb.2024.1392473 (PMC11039966; doi:10.3389/fmicb.2024.1392473)
Supplement: Supplementary file 1 [file Data_Sheet_1.zip › Supplementary Materials/MR plots of saliva/Tonsillitis/s__Porphyromonas_sp000467855_mgs_2773/forest.pdf]

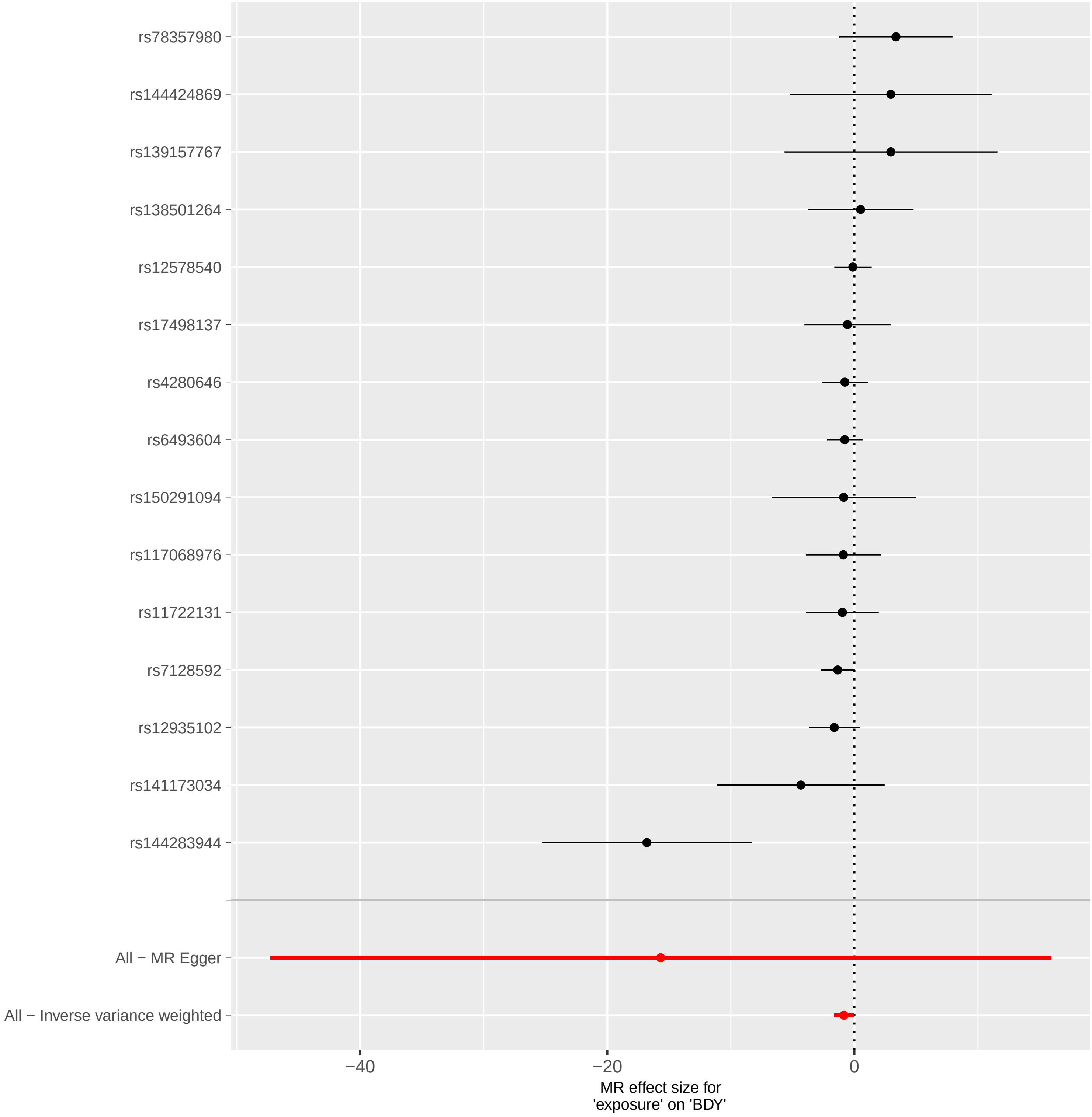
rs78357980
rs144424869
rs139157767
rs138501264
rs12578540
rs17498137
rs4280646
rs6493604
rs150291094
rs117068976
rs11722131
rs7128592
rs12935102
rs141173034
rs144283944
All − MR Egger
All − Inverse variance weighted
−40
−20
0
MR effect size for
'exposure' on 'BDY'
